# Posterior fossa ependymoma H3 K27-mutant: an integrated radiological and histomolecular tumor analysis

**DOI:** 10.1186/s40478-022-01442-4

**Published:** 2022-09-14

**Authors:** Cassandra Mariet, David Castel, Jacques Grill, Raphaël Saffroy, Volodia Dangouloff-Ros, Nathalie Boddaert, Francisco Llamas-Guttierrez, Céline Chappé, Stéphanie Puget, Lauren Hasty, Fabrice Chrétien, Alice Métais, Pascale Varlet, Arnault Tauziède-Espariat

**Affiliations:** 1 Department of Pediatric Oncology, Gustave Roussy, Université Paris-Saclay, 94805 Villejuif, France; 2 U981, Molecular Predictors and New Targets in Oncology, INSERM, Gustave Roussy, Université Paris-Saclay, 94805 Villejuif, France; 3 Department of Biochemistry and Oncogenetic, Paul Brousse Hospital, 94804 Villejuif, France; 4 Pediatric Radiology Department, Hôpital Necker Enfants Malades, AP-HP, Paris, France; 5 Université Paris Cité, UMR 1163, Institut Imagine and INSERM U1299, Paris, France; 6 Department of Pathology, Ponchaillou Hospital, Rennes, France; 7 Department of Oncology, Ponchaillou Hospital, Rennes, France; 8 Department of Pediatric Neurosurgery, Necker Hospital, APHP, Université Paris Descartes, Sorbonne Paris Cite, 75015 Paris, France; 9 Department of Neuropathology, GHU Paris-Psychiatrie Et Neurosciences, Sainte-Anne Hospital, 1, rue Cabanis, 75014 Paris, France; 10 Inserm, UMR 1266, IMA-Brain, Institut de Psychiatrie Et Neurosciences de Paris, Paris, France

**Keywords:** Posterior fossa, Ependymoma, Histones, DNA-methylation

## Abstract

Posterior fossa group A ependymomas (EPN_PFA) are characterized by a loss of H3 K27 trimethylation due to either *EZHIP* overexpression or H3 p.K27M mutation, similar to H3 K27-altered diffuse midline gliomas (DMG), but in reverse proportions. Very little data is available in the literature concerning H3 K27M-mutant EPN_PFA. Here, we retrospectively studied a series of nine pediatric tumors initially diagnosed as H3 K27M-mutant EPN_PFA to compare them to EZHIP-overexpressing EPN_PFA in terms of radiology, follow-up, histopathology, and molecular biology (including DNA-methylation profiling). Seven tumors clustered within EPN_PFA by DNA-methylation analysis and t-distributed stochastic neighbor embedding. Among the two remaining cases, one was reclassified as a DMG and the last was unclassified. H3 K27M-mutant EPN_PFA cases were significantly older than their counterparts with an EZHIP overexpression. Radiological and histopathological central review of our seven H3 K27M-mutant EPN_PFA cases found them to be similar to their counterparts with an EZHIP overexpression. Sequencing analyses revealed *HIST1H3B* (n = 2), *HIST1H3C* (n = 2), *H3F3A* (n = 1), and *HIST1H3D* (n = 1) K27M mutations (no sequencing analysis available for the last case which was immunopositive for H3K27M). Consequently, *HIST1H3C/D* mutations are more frequently observed in EPN_PFA than in classic pontine DMG, H3K27-mutant. Overall survival and event-free survival of EZHIP-overexpressing and H3 K27M-mutant EPN_PFA were similar. After surgery and radiation therapy, 5/7 patients were alive at the end of the follow-up. In summary, the diagnosis of EPN_PFA must include tumor location, growth pattern, Olig2 expression, and DNA-methylation profiling before it can be differentiated from DMG, H3 K27-altered.

**Supplementary Information:**

The online version contains supplementary material available at 10.1186/s40478-022-01442-4.

## Introduction

In the central nervous system (CNS), the loss of H3 p.K27me3 expression generally constitutes the hallmark of two different tumor types: diffuse midline glioma (DMG), H3 K27-altered and posterior fossa ependymoma, subtype A (EPN_PFA) [6, 15]. This loss is due to an overexpression of EZHIP or to K27M mutations in histone H3 genes (mostly *H3F3A* and *HIST1H3B*), inhibiting the methyltransferase activity of the Polycomb Repressive Complex 2 (PRC2) [5]. EZHIP overexpression and H3 K27M mutations are mutually exclusive and are encountered in reverse proportions: 3% versus 97% and 96% versus 4%, respectively in DMG and EPN [15]. K27M histones’ genes mutations have also been exceptionally reported in other tumor types, such as one desmoplastic/nodular medulloblastoma, SHH-activated [7], one embryonal tumor with multilayered rosettes of the posterior fossa (harboring also a *DICER1* mutation) [20], and *BRAF*-mutant midline gangliogliomas [9, 10, 14]. Because of these potential histomolecular diagnostic pitfalls, the Consortium to Inform Molecular and Practical Approaches to CNS Tumor Taxonomy—Not Official WHO (cIMPACT-NOW), and the World Health Organization (WHO) classification of tumors of the CNS clarified the definition of the DMG, H3 K27-altered, in which each term is mandatory: diffuse (infiltrating the neuropil background, particularly on neurofilament staining), midline (including thalamus, brainstem and spinal cord), and glioma (with the expression of glial markers, particularly Olig2) [11, 12]. Thus, a tumor previously initially diagnosed as a brainstem DMG, EZHIP-overexpressing, was reclassified as an EPN_PFA by DNA-methylation analysis [16]. Interestingly, this tumor met two of the three essential DMG diagnostic criteria (midline location and infiltrative, but did not express Olig2) [16]. DMG may present a wide variety of morphologies including ependymoma-like features [3, 12, 18]. Very few histopathological and epigenetic data are available for previously described EPN_PFA with H3 K27M mutation (22 published cases) [8, 13, 15, 17, 21]. To explore this issue, we investigated a cohort of 147 EPN_PF to detect H3 K27M-mutant cases in order to better characterize them in terms of radiology, follow-up, histopathology, and molecular biology (including DNA sequencing and DNA-methylation profiling).

## Methods

### Sample collection

Tumor samples of EPN_PF were provided by the consultation archive database (1993–2021) from the department of neuropathology at GHU-Paris Psychiatry and Neurosciences, Sainte-Anne Hospital. Thereafter, we screened the national French neuropathological network database and searched for other cases presenting the same histopathology.

This study was approved by our local ethics committee. Written informed consent for clinical and imaging investigations and molecular analysis was obtained from all patients enrolled in this study.

### Clinical and radiological data

Patient characteristics and clinical data retrieved from hospital records included: sex, age at presentation, and medical history. The central radiological review of preoperative magnetic resonance imaging (MRI) was performed by two senior neuroradiologists (VDR and NB). The following features were evaluated: location (tumor epicenter), size, volume, signal in T1-weighted sequence, T2-weighted sequence, susceptibility imaging, diffusion and apparent coefficient diffusion map (ADC), contrast enhancement, contours, presence of cysts, necrosis, and perfusion parameters.

### Central histopathological review and immunohistochemistry

The central pathology review was performed conjointly by two neuropathologists (ATE and PV). According to the last WHO classification, no grading was applied. A representative paraffin block was selected for each case. Unstained 3-μm-thick slides of formalin-fixed, paraffin-embedded tissues were obtained and submitted for immunostaining. The following primary antibodies were used: Glial Fibrillary Acidic Protein (GFAP) (1:200, clone 6F2, Dako, Glostrup, Denmark), epithelial membrane antigen (1:200, clone GM008, Dako, Glostrup, Denmark), Olig2 (1:3000, clone C-17, Santa Cruz Biotechnology, Dallas, USA), Neurofilament Protein (1:100, clone 2F 11, Dako, Glostrup, Denmark), H3K27me3 (1:2500, polyclonal, Diagenode, Liege, Belgium), EZHIP (1:75, polyclonal, Sigma-Aldrich, Bromma, Sweden) and H3K27M (1:5000, clone EPR18340, Abcam, Cambridge, United Kingdom). External positive and negative controls were used for all antibodies.

### Detection of H3 K27M mutation by MassArray and Sanger Sequencing

Genomic DNA was extracted from formalin-fixed and paraffin-embedded (FFPE) tissue. K27M mutations in *H3F3A*, *HIST1H3B* and *HIST1H3C* were detected using the Massarray iPlex technology (Agena Bioscience). The Massarray iPlex procedure involves a three-step process consisting of the initial PCR reaction, inactivation of unincorporated nucleotides by shrimp alkaline phosphatase and a single-base primer extension. The products are then nano-dispensed onto a matrix-loaded silicon chip (SpectroChipII, Agena Bioscience) and finally, the mutations are detected by MALDI–TOF (matrix-assisted laser desorption-ionization–time of flight) mass spectrometry. Data analysis was performed using MassArrayTyper Analyzer software 4.0.4.20 (Agena Bioscience). Negative cases with sufficient gDNA were further submitted for a whole exome analysis (WES) at Integragen (Evry, France) and analysed as previously published [5]. When this technique was not contributive, genomic DNA was extracted from fresh-frozen or formalin-fixed and paraffin-embedded (FFPE) tissue. K27M mutations in *H3F3A*, *HIST1H3B* and *HIST1H3C* were detected by direct Sanger sequencing of PCR amplified products from tumor genomic DNA as previously published [6]. Negative cases with sufficient gDNA were further submitted to a whole exome analysis (WES) at Integragen (Evry, France) and analysed as previously published [5].

### DNA methylation array processing and copy number profiling

DNA extraction from FFPE was performed using the QIAamp DNA FFPE Tissue Kit and the Qiacube (QIAGEN, Hilden, Germany) according to the manufacturer’s instructions. 250 to 500 ng of DNA were extracted from each tissue sample. Kits used for bisulfite conversion and reparation were the Zymo EZ DNA methylation kit and ZR-96 DNA Clean and Concentrator-5 (Zymo Research, Irvine, CA) and bisulfite DNA was processed using the Illumina Infinium HD FFPE DNA Restore kit and Infinium FFPE QC kit (Illumina, San Diego, CA). The DNA was then processed using the Illumina Infinium HumanMethylation EPIC Bead-Chip array (Illumina, San Diego, CA) according to the manufacturer’s instructions. The iScan control software was used to generate raw data files from the BeadChip in IDAT format and analyzed using GenomeStudio version 2.0 (Illumina, San Diego, CA). The following filtering criteria were applied: removal of probes targeting the X and Y chromosomes, removal of probes containing single nucleotide polymorphisms (dbSNP132 Common) within five base pairs of and including the targeted CpG site, and removal of probes not mapping uniquely to the human reference genome (hg19), allowing for one mismatch [4]. The raw IDAT files were uploaded to www.molecularneuropathology.org for supervised analysis using the Random Forest methylation class prediction algorithm, as previously described [4]. All computational analyses were performed in R version 3.3.1 (R Development Core Team, 2016; https://www.R-project.org). Copy-number variation analyses from 450 k and EPIC methylation array data were performed using the conumee Bioconductor package version 1.12.0. Raw signal intensities were obtained from IDAT-files using the minfi Bioconductor package version 1.21.4. Illumina EPIC samples and 450 k samples were merged to a combined data set by selecting the intersection of probes present on both arrays (combineArrays function, minfi). Each sample was individually normalized by performing a background correction (shifting of the 5% percentile of negative control probe intensities to 0) and a dye-bias correction (scaling of the mean of normalization control probe intensities to 10,000) for both color channels. Subsequently, a correction for the type of material tissue (FFPE/frozen) and array type (450 k/EPIC) was performed by fitting univariable, linear models to the log2-transformed intensity values (removeBatchEffect function, limma package version 3.30.11). The methylated and unmethylated signals were corrected individually. Beta-values were calculated from the retransformed intensities using an offset of 100 (as recommended by Illumina). All samples were checked for duplicates by pairwise correlation of the genotyping probes on the 450 k/850 k array. To perform unsupervised non-linear dimension reduction, the remaining probes after standard filtering were used to calculate the 1-variance weighted Pearson correlation between samples. The resulting distance matrix was used as input for t-SNE analysis (t-distributed stochastic neighbor embedding; Rtsne package version 0.13). The following non-default parameters were applied: theta = 0, pca = F, max_iter = 20,000 perplexity = 10. The t-SNE analysis was performed and compared with the genome-wide DNA methylation profiles from selected reference groups of the brain tumor reference cohort [4].

## Results

### Of the 147 tumors classified as EPN_PF, 5% of them exhibited a H3 K27M immunopositivity

**Fig. 1:**
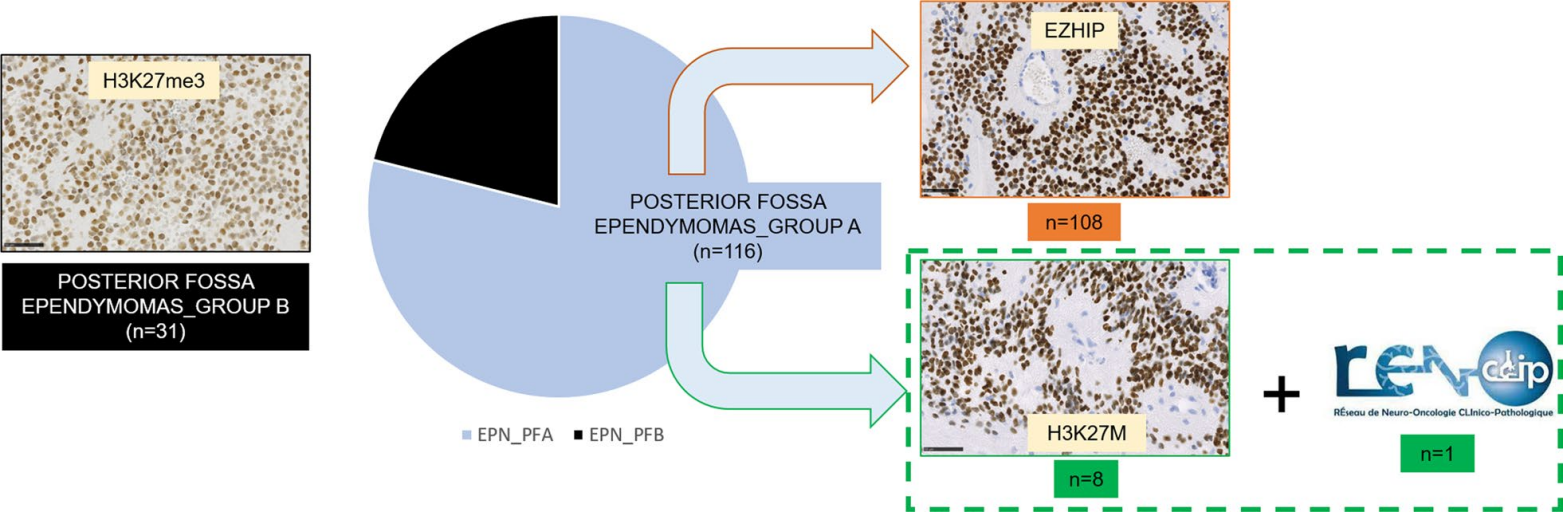
Cohort characteristics. Based on H3K27me3 immunohistochemistry, 116 cases were classified as EPN_PFA showing a loss of immunoexpression, and 31 were EPN_PFB with a conserved H3K27me3 expression. From the 116 EPN_PFA, 108 were immunopositive for EZHIP and eight cases were immunopositive for the H3K27M mutation. From the national French Neuropathological Reference network (RENOCLIP-LOC) database, we found one additional pediatric case of EPN_PFA with H3K27M immunopositivity. Scale bars represent 50 µm

147 cases were retrieved and reviewed by two pathologists (PV and ATE). 131 (89%) patients were children and 16 (11%) were adults. The median age of the whole cohort was 8-years-old (aged from 0 to 72-years-old). The female to male ratio was 0.9 (71 females and 76 males). Based on immunohistochemistry, 116 cases were EPN_PFA (all pediatrics) showing a loss of H3K27me3 immunoexpression, and 31 were EPN_PFB (15 children and 16 adults) with a conserved H3K27me3 expression. From the 116 EPN_PFA, 108 (93%) were immunopositive for EZHIP and eight cases (7%, 5% of the whole cohort) were immunopositive for the H3K27M mutation. From the national French Neuropathological Reference network (RENOCLIP-LOC) database, we found one additional pediatric case of EPN_PFA with H3K27M immunopositivity (Fig. 1).

### Posterior fossa tumors with ependymal/subependymal features, H3 K27M-positive encompass different subgroups with distinct clinical outcomes

**Fig. 2:**
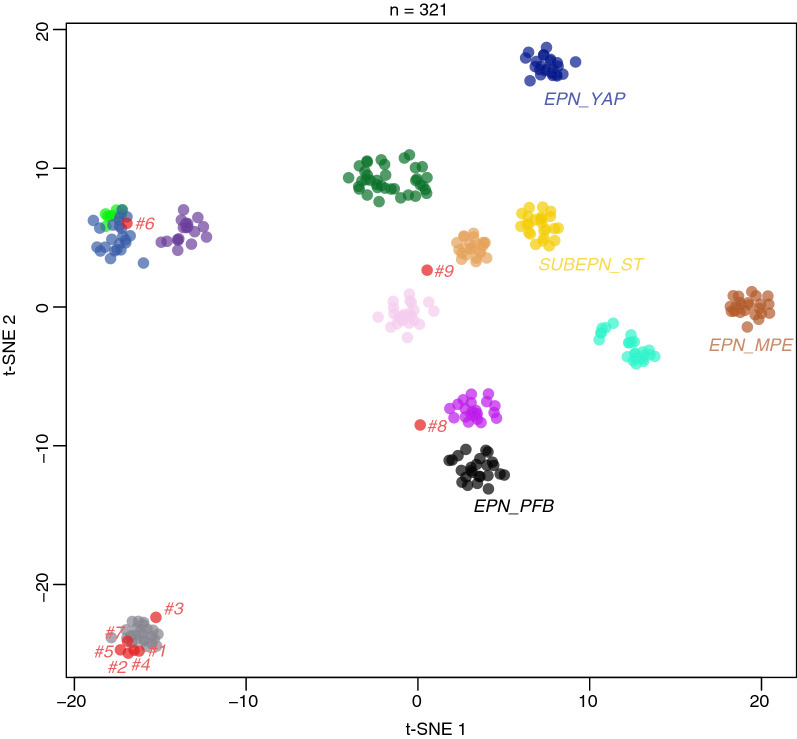
t-Distributed Stochastic Neighbor Embedding plot (t-SNE) analysis of our cases compared with reference samples from the Heidelberg cohort. DMG_EGFR: Diffuse midline glioma, *EGFR-*altered; DMG_EZHIP: Diffuse midline glioma, EZHIP-overexpressing; DMG_K27: Diffuse midline glioma, H3K27M-mutant; EPN_PFA: Ependymoma of the posterior fossa, subtype A; EPN_PFB: Ependymoma of the posterior fossa, subtype B; EPN_MPE: Myxopapillary ependymoma; EPN_SPINE: Ependymoma of the spine; EPN_YAP: Ependymoma, *YAP1*-fusion positive; EPN_ZFTA: Ependymoma, *ZFTA-*fusion positive; GG: Ganglioglioma; SUBEPN_PF: Subependymoma of the posterior fossa; SUBEPN_SPINE: Subependymoma of the spine; SUBEPN_ST: Supratentorial subependymoma

We subjected these nine H3 K27M EPN_PFA cases to DNA-methylation profiling (with the v12.5 of the DKZF classifier of CNS tumors), and 5/9 cases presented a high calibrated score (≥ 0.9), classified as EPN_PFA, subclass 1f (#1, 2, 4, 5, and 7). We also performed a t-SNE analysis on the nine cases to better classify tumors with low calibrated scores (< 0.9) (Fig. 2) and integrate it with clinical, radiological and histomolecular findings.

Accordingly, six cases (#1, 2, 3, 4, 5 and 7) were classified by DNA-methylation profiling as EPN_PFA (Fig. 2, Additional file 1: Supplementary Fig. 1 for copy number variations). They were located in the fourth ventricle. All but one had a K27M mutation of one of the genes encoding for the H3.1 protein (*HIST1H3B* in two cases, *HIST1H3C* in two cases, and *HIST1H3D* in one case). For the last case (#2), DNA sequencing analyses failed to reveal any mutation of histones’ genes due to the poor quality of the remaining DNA. At the end of follow-up, 5/6 patients were alive (median follow-up of 68 months) (Table 1).

**Table 1:** Summary of clinicopathological features of the PFA_EPN, H3K27M-mutant including cases from the literature

	Author Reference	Age (years)/Sex	WHO Grade	Mutation	1q gain/6q loss (CNV)	Methylation class (calibrated score)	Integrated diagnosis	Resection	Adjuvant therapy	Outcome
*1*	Current study	5/M	ND*	*HIST1H3B*	Absent/absent	PFA-EPN 1f (0.99)	PFA-EPN	GTR	Radiotherapy	Spinal relapse at 16 months Death at 50 months
*2*		5/F	ND*	NA	Absent/present	PFA-EPN 1f (0.99)	PFA-EPN	Subtotal resection	Radiotherapy	Spinal relapse at 6 months Alive at 50 months
*3*		10/F	ND*	*HIST1H3C*	Absent/absent	PFA-EPN 1a (0.23)	PFA-EPN	GTR	Radiotherapy	Spinal relapse at 107 months Alive at 130 months
*4*		12/M	ND*	*HIST1H3B*	Absent/absent	PFA-EPN 1f (0.95)	PFA-EPN	GTR	Radiotherapy	Relapse-free Alive at 101 months
*5*		9/F	ND*	*HIST1H3C*	Present/absent	PFA-EPN 1f (0.99)	PFA-EPN	Subtotal resection	Radiotherapy	Relapse at 79 months Alive at 82 months
*7*		8/M	ND*	*HIST1H3D*	Absent/absent	PFA-EPN 1f (0.99)	PFA-EPN	GTR	Radiotherapy	Alive at 3 months
*8*		1.5/M	ND*	*H3F3A*	Absent/absent	PFA-EPN 1a (0.45)	PFA-EPN	GTR	Radiotherapy Everolimus Avastin	Death at 88 months
*10*	Gessi [8]	1.5/F	3	*H3F3A*	NA/NA	PFA-EPN	PFA-EPN	Biopsy	E-HIIT 2000 R	Relapse free Alive at 6 months
*11*		6/F	3	*HIST1H3C*	NA/NA	PFA-EPN	PFA-EPN	GTR	E-HIIT 2000 AB4	Spinal relapse at 19 months Alive at 49 months
*12*	Nambirajan [13]	2/F	2	*H3F3A*	Absent/NA	NA	NA	GTR	Radiotherapy	Relapse at 3 months Alive at 24 months
*13*	Ryall [17]	7/M	3	*H3F3A*	Absent/absent	NA	NA	GTR	Radiotherapy	Local relapse at 18 months Death at 24 months
*14*	Tanrikulu [19]	1.5/M	3	NA	NA/NA	NA	NA	GTR	Chemotherapy	Relapse-free Alive at 86 months
*15*	Yao [21]	2/M	1	*H3F3A*	NA/NA	NA	NA	No GTR	No	Relapse-free Alive at 60 months
*15*		43/M	1	*H3F3A*	NA/NA	NA	NA	No GTR	No	Relapse and death at 37 months
*16*		13/M	1	*H3F3A*	NA/NA	NA	NA	No GTR	No	Relapse-free Alive at 40 months
*17*		20/M	1	*H3F3A*	NA/NA	NA	NA	No GTR	No	Relapse-free Alive at 3 months
	Pajtler [15]	NA	NA	*HIST1H3B* (n = 4)	NA/NA	Ependymoma 1f (9/13) Ependymoma 1a, 1d, 1e	PFA-EPN	NA	NA	NA
				*HIST1H3C* (n = 7)	NA/NA		PFA-EPN			
				*H3F3A* (n = 2)	NA/NA	Ependymoma 1a	PFA-EPN			

CNV: copy number variation; EPN: ependymoma; F: female; GTR: Gross total resection; M: male; NA: not available; ND: not detailed; PFA: posterior fossa group A; WHO: World Health Organization

^*^All cases presented high-grade features

**Fig. 3:**
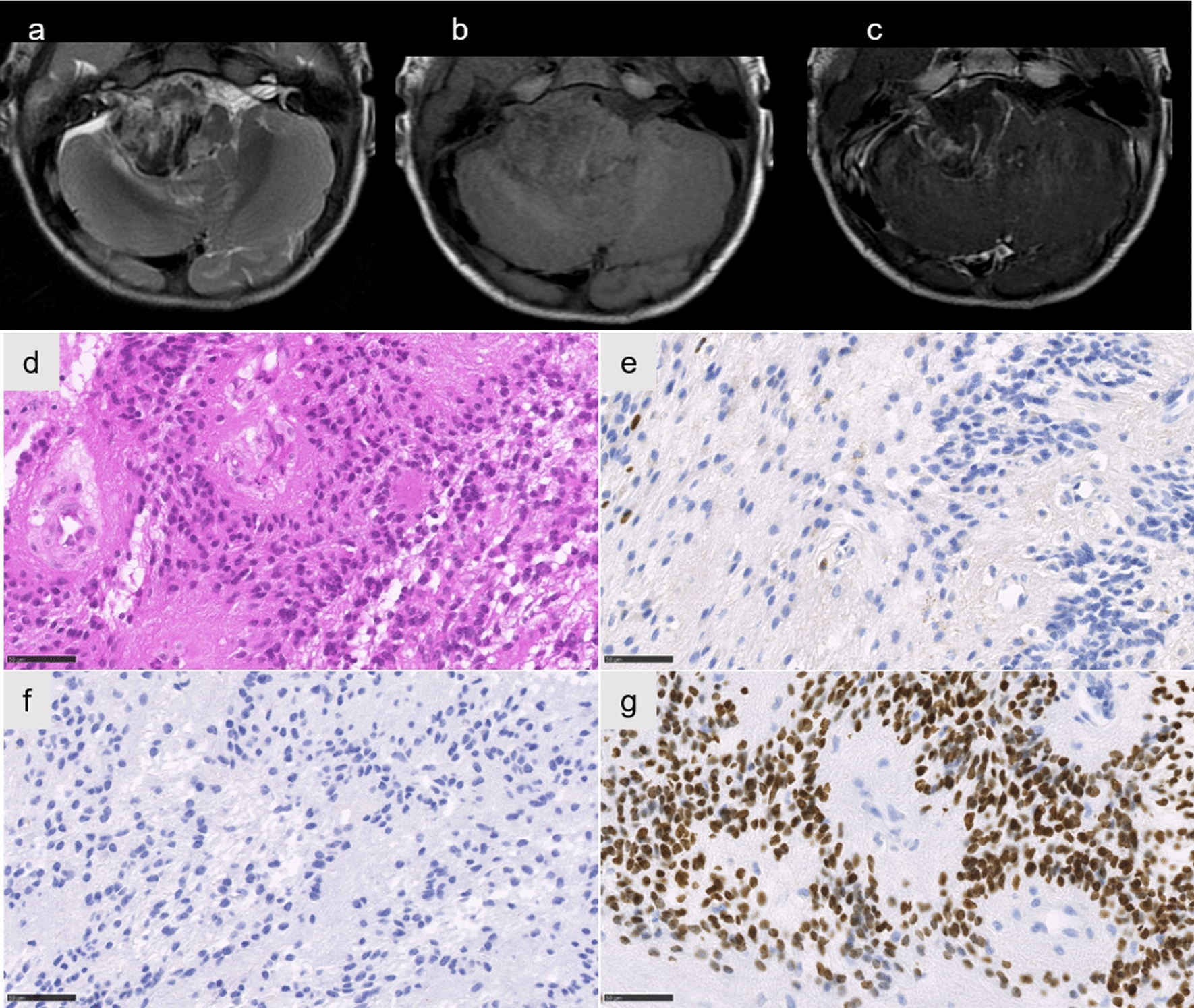
Radiological and histopathological features of case #8. Axial T2-weighted image showing a circumscribed hemorrhagic lesion of the right cerebellopontine angle (**a**), with a low signal on T1-weighted images (**b**), and a peripheral enhancement on T1-weighted image with gadolinium injection (**c**). Ependymal features with pseudorosettes (**d** HPS, magnification 400$\times$), associated with no immunoreactivity for Olig2 (**e** magnification 400$\times$). Neurofilament immunostaining confirming the solid pattern (**f** magnification 400$\times$). Immunoexpression of H3K27M in all tumor cells (**g** magnification 400$\times$). Scale bars represent 50 µm. HPS: Hematoxylin Phloxin Saffron

One case (#8) was classified by the v12.5 of the brain classifier as EPN_PFA, subclass 1a (but with a low calibrated score of 0.45), but the t-SNE analysis did not support this data. The tumor did not cluster within a methylation class, but was in proximity to posterior fossa subependymomas (Fig. 2). This case concerned a 1-year-old boy presenting recent (< 1 month) symptoms of intracranial hypertension. The MRI revealed a voluminous and well-circumscribed tumor arising from the right cerebellopontine angle (CPA) with spontaneous hemorrhage and contrast enhancement (Fig. 3a–c). Histopathologically, the tumor presented a solid growth pattern and immunonegativity for Olig2 (Fig. 3d–f). The expression of H3K27M by tumor cells (Fig. 3g) was confirmed by DNA sequencing analysis showing a *HIST1H3B* K27M mutation. This patient died suddenly after 88 months without evidence of relapse or progression, following adjuvant treatments by radiation therapy and chemotherapy. Based on the WHO classification, the integrated diagnosis was EPN_PFA with H3K27M mutation.

**Fig. 4:**
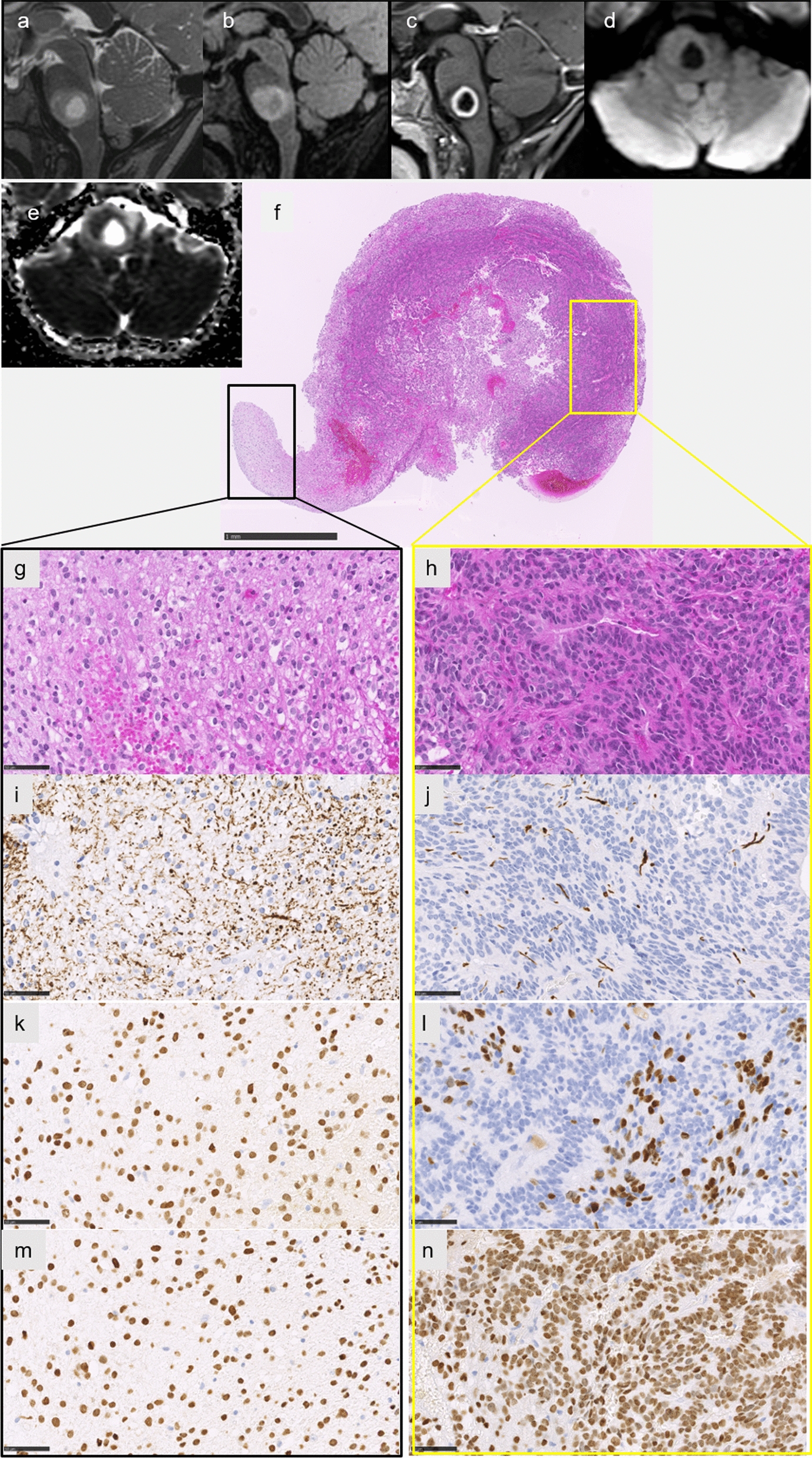
Radiological and histopathological features of case #6. Sagittal T2-weighted image (**a**) and FLAIR image (**b**) showing an infiltrative lesion of the pons with a central liquid part consistent with necrosis, with a peripheral enhancement of the necrosis on T1-weighted image with gadolinium injection (**c**), and intermediate signal on diffusion-weighted image (**d**) with partially restricted diffusion on apparent diffusion coefficient map (**e**). The biopsy highlighted two different histopathological components (**f** HPS, magnification × 50). The glial diffuse component (**g** HPS, magnification 400$\times$), associated with an ependymal component with rosettes (**h** HPS, magnification 400$\times$). Neurofilament immunostaining confirming the infiltrative pattern in the glial component (**i** magnification 400$\times$) and a more circumscribed pattern in the ependymal component (**j** magnification 400$\times$). Diffuse immunopositivity for Olig2 in the glial component (**k** magnification × 400), and partial expression of Olig2 in the ependymal component (**l** magnification × 400). Diffuse immunoexpression of H3K27M in both tumoral components (**m–n** magnification 400$\times$). Scale bars represent 1 mm (**f**) and 50 µm (**g–n**). HPS: Hematoxylin Phloxin Saffron

Case #6 was classified by the v12.5 of the brain classifier as DMG, H3K27M-mutant, but with a calibrated score of 0.84. This data was confirmed by the t-SNE analysis where it perfectly clustered with DMG, H3 K27-altered, subtype H3 K27-mutant or EZHIP overexpressing (Fig. 2). This case concerned a 7-year-old boy with recent (< 3 weeks) symptoms of cranial nerve palsy and hemiparesis revealing a pontine tumor (Fig. 4a, b). Radiologically, the tumor was intrinsic to the pons, infiltrative, showed a high signal on T2-weighted images, and had focal necrosis with peripheral enhancement (Fig. 4b, c). Diffusion was restricted in one part of the tumor (Fig. 4d, e). Morphologically, this tumor was composed of two distinct glial histopathological components: one solid with ependymal features (pseudorosettes and true rosettes) and a weak expression of Olig2 and one infiltrative component with an Olig2 expression (Fig. 4f–l). Both components expressed H3K27M and an *H3F3A* K27M mutation was confirmed by DNA sequencing analysis (Fig. 4m–n). After the biopsy, the patient received radiation therapy and chemotherapy with Dasatinib, but died 11 months after the initial diagnosis (Additional file 2: Supplementary Table 1). The integrated diagnosis of this case was DMG, H3 K27-altered with ependymal features.

**Fig. 5:**
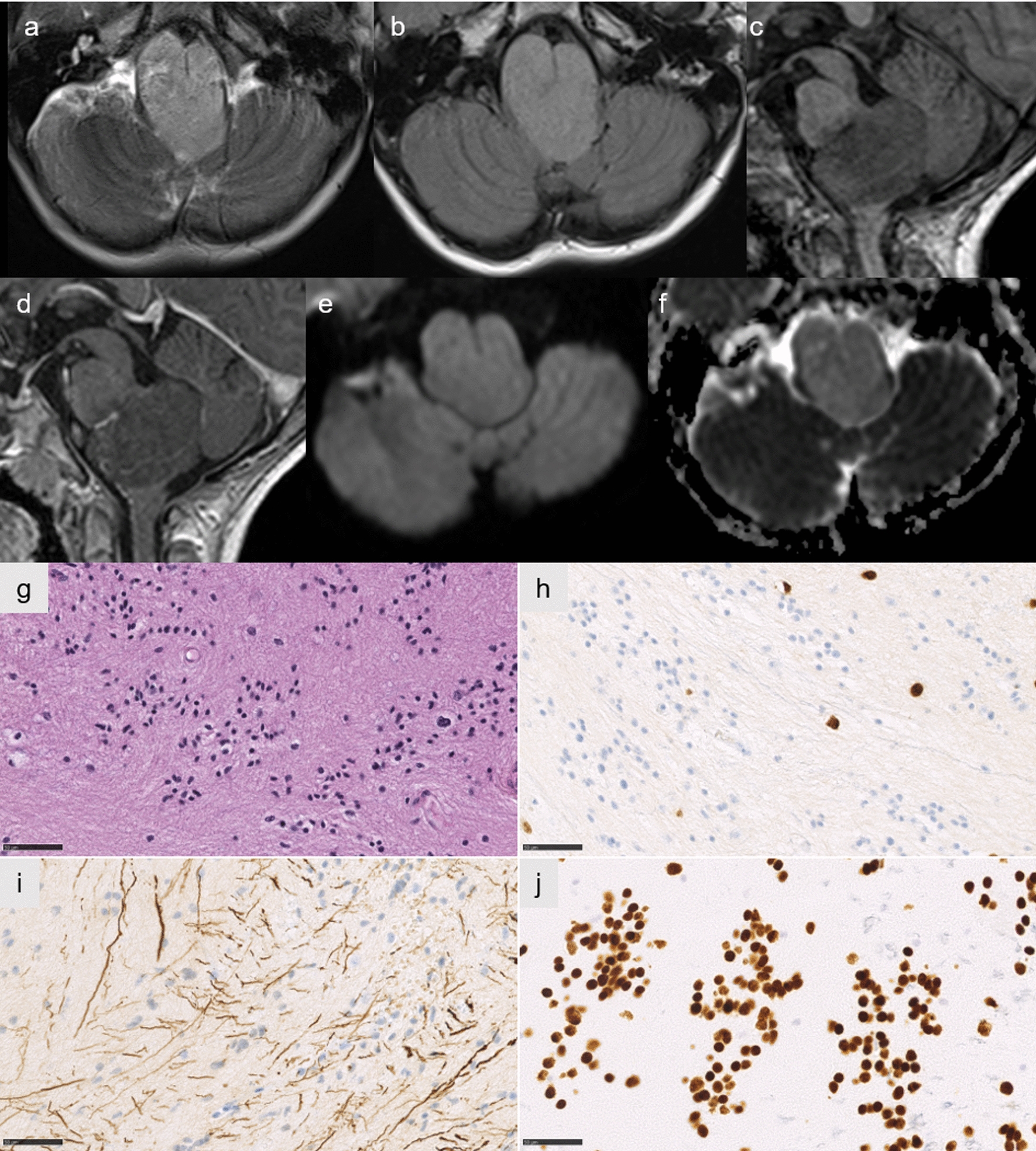
Radiological and histopathological features of case #9. Axial T2-weighted and FLAIR image showing a circumscribed lesion of the *medulla oblongata* (**a,b**), without enhancement on sagittal T1-weighted image with gadolinium injection (**c** before injection,** d** after injection), and low signal on diffusion weighted image (**e**) with increased diffusion on apparent diffusion coefficient map (**f**). A paucicellular tumor with subependymal features (**g** HPS, magnification 400$\times$). No immunoreactivity for Olig2 except in residual glial cells (**h** HPS, magnification 400$\times$). Neurofilament immunostaining showing an infiltrative pattern (**i** magnification 400$\times$). Diffuse immunoexpression of H3K27M in tumor cells (**j** magnification 400$\times$). Scale bars represent 50 µm. HPS: Hematoxylin Phloxin Saffron

The last case (#9) was classified by the v12.5 of the brain classifier as DMG, H3K27M-mutant (but with a low calibrated score of 0.54), however the t-SNE analysis did not confirm this data. The tumor did not cluster within a methylation class (Fig. 2). This case concerned an 8-year-old boy who did not present a short medical history but headaches and oculomotor nerve palsy lasting nine months before exhibiting rapidly progressing brainstem dysfunction symptoms (trouble swallowing, hemiparesis). The MRI showed a well-circumscribed tumor limited to the *medulla oblongata* with a high T2-weighted signal, without infiltration of the spine (Fig. 5a–c). There was no enhancement after injection of gadolinium (Fig. 5d). There was no restriction of the diffusion (high apparent diffusion coefficient) (Fig. 5e, f). Histopathologically, the tumor was paucicellular with subependymal features and without Olig2 immunopositivity (Fig. 5g, h). However, the tumor showed an infiltrative growth pattern (Fig. 5i). All tumor cells expressed H3K27M, which was confirmed by DNA sequencing as a K27M *H3F3A* mutation (Fig. 5j). After the biopsy, the patient was treated by radiation therapy and chemotherapy with Everolimus and Bevacizumab, but died eight months after the initial diagnosis (Additional file 2: Supplementary Table 1). Based on the WHO classification and because of discrepancies between histopathology and epigenetic results, no integrated diagnosis was made.

### No differences in terms of histopathology and outcome between EPN_PFA EZHIP-overexpressing and their counterpart with H3K27M mutation

**Fig. 6:**
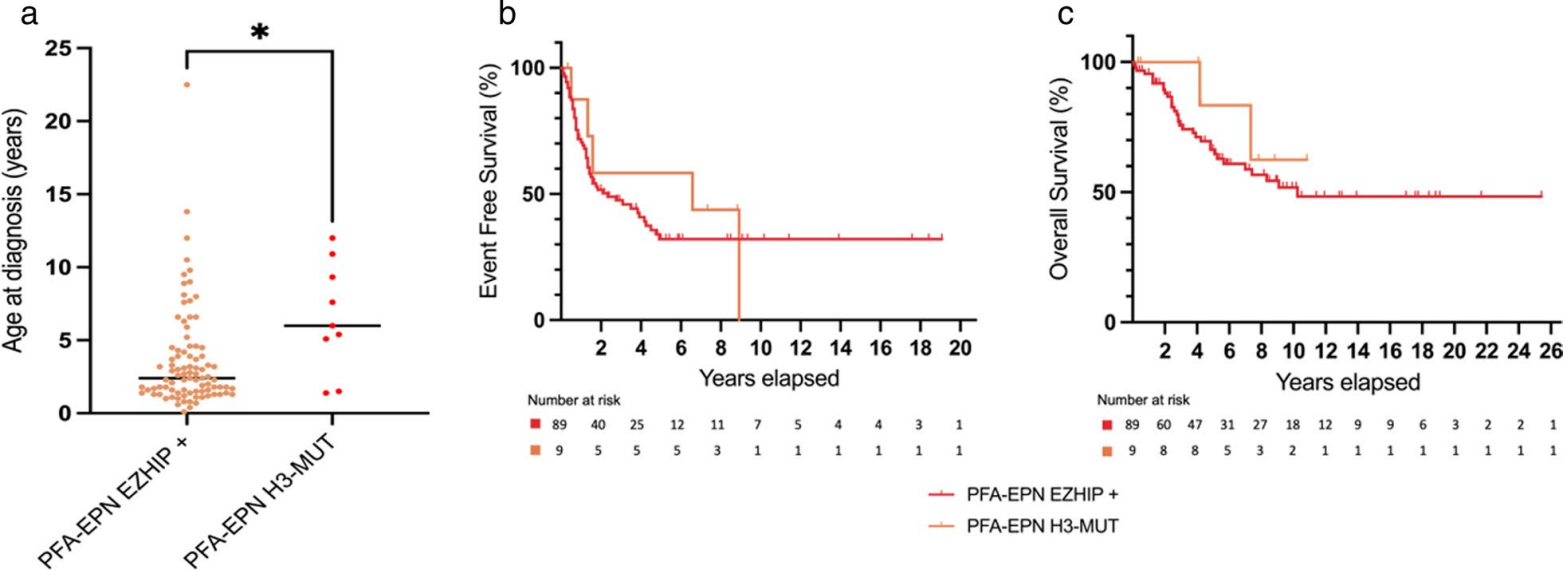
Age distribution and outcome of PFA_EPN, EZHIP-overexpressing and H3K27M-mutant of our cohort (n = 7) and cases from the literature with available methylation data (n = 2, cases #10 and #11). Median age for EPN_PFA, H3K27M-mutant of 6 years (1.4–12) *versus* 2.4 years for EPN_PFA, EZHIP-overexpressing (0.1–22.5). Statistical significance was calculated with Mann–Whitney test (median, *p value 0.0168, n = 98) (**a**). Kaplan–Meier survival estimates of EFS and OS: no significant difference between EPN_PFA, EZHIP-overexpressing (red, n = 89, median EFS 28 months, median OS 123 months) and H3K27M-mutant (orange, n = 9, median EFS 79 months, median OS not reached), log rank test, *p* value 0.67 and 0.41 respectively (**b**, **c**). DMG: diffuse midline gliomas; EPN: ependymomas; EFS: event-free survival; OS: overall survival; PF: posterior fossa

After integrated diagnoses, the results of our cohort of confirmed EPN_PFA (n = 7) showed that patients with H3K27M-mutant EPN_PFA (median age of 6 years) were significantly older than EZHIP-overexpressing EPN_PFA patients (median age of 2.4 years) (p = 0.0168) (Fig. 6a), as opposed to DMG patients [4].

Radiologically, EPN_PFA with an H3K27M mutation presented as a well-delineated solid tumor located in the fourth ventricle except for one case located in the cerebellopontine angle (Additional file 1: Supplementary Fig. 2). Peritumoral edema was found in approximately half of cases. Calcifications were present in the 3/3 cases having available CT. By MRI, tumors displayed intense heterogeneous enhancement after gadolinium chelate injection. A lateral extension of the tumors via the foramen of Luschka was present as in EPN_PFA EZHIP-overexpressing whereas their H3K27M-mutant counterparts did not invade the posterior fossa foramina.

Histopathologically, no differences were found between EPN_PFA EZHIP-overexpressing and EPN_PFA H3K27M-mutant (Additional file 1: Supplementary Fig. 2). All EPN_PFA H3K27M-mutant showed the histopathological features for EPN, including pseudorosettes and rosettes. All of the tumors were well-circumscribed using neurofilament staining. Calcifications were observed in 5/7 cases which are rare in DMG, H3 K27-altered. High-grade features, including prominent mitotic activity (7/7 cases), necrosis (5/7 cases) and microvascular proliferation (5/7 cases) were present. Immunohistochemical analyses showed classical features of EPN with a diffuse expression of GFAP, a focal or absent expression of Olig2 and dot-like immunopositivity for EMA in all cases.

Overall survival (OS) and event-free survival (EFS) of EZHIP-overexpressing compared to H3K27M-mutant EPN_PFA seem to present no statistical difference (Fig. 6b, c). To note, the case classified as DMG with ependymal features, H3K27M-mutant (case #6) presented an OS similar to DMG, rather than EPN.

## Discussion

This series constitutes the first attempt to perform a comprehensive clinico-radiological, histopathological, genetic and epigenetic characterization of EPN_PFA H3 K27M-mutant. Our cohort highlights the difficulties encountered when reaching this diagnosis and the potential overlaps with DMG, H3 K27-altered. The DNA-methylation profiling showed that H3 K27M-mutant posterior fossa tumors with ependymal/subependymal features may represent different epigenetic subgroups, confirming that the DNA-methylation profile is not dominated by consequences of H3 mutations but possibly by the cell of origin. Our results confirm that DMG, H3 K27-altered, may present morphological ependymal features [18]. These results reinforce the essential criteria of the WHO classification of tumors of the CNS for DMG, H3 K27-altered [11, 12]. However, our results indicate that diagnostic criteria of EPN_PFA and DMG, H3 K27-altered do not permit the classification of all posterior fossa tumors H3K27M-mutant. Indeed, one of our cases (#9) was not classified using the DNA-methylation profile. Further cases are needed to better characterize and clarify these difficult cases having overlapping similarities to EPN, subependymomas and DMG. Because most reported cases lack DNA-methylation profiles and the main histopathological criteria (such as the growth pattern and Olig2 expression), the literature review remains difficult to analyze but the hypothesis of a DMG with ependymal/subependymal features could be proposed [2, 13, 17, 19, 21]. Indeed, contrary to DMG, H3 K27-altered, *H3F3A* mutations do not represent the main molecular event in EPN_PFA, H3K27M-mutant, in which H3.1 gene mutations are overrepresented [6]. Our results compiled with those from the literature (including only articles with DNA-methylation analyses) show that H3.1 gene K27M mutations represent 85% of mutations (17/20 cases) encountered in EPN_PFA including: *HIST1H3C* (n = 10), *HIST1H3B* (n = 6), and herein, for the first time (including in the DMG population), *HIST1H3D* (n = 1) [8, 15]. The three remaining cases (15%) harbored an *H3F3A* K27M mutation [8, 15]. After integrated diagnoses, the frequency of H3K27M mutations in EPN_PFA of our cohort (7/116, 6%) is slightly higher than previous series (1 to 4% among the cohorts) [13, 15, 17]. Our results suggest that this PFA subgroup may differ from its overexpressing EZHIP counterpart, in terms of age and location. All but one tumor (located in the cerebellopontine angle) were midline and located in the fourth ventricle, which is less frequently observed in EPN_PFA with EZHIP-overexpression [1]. A histopathological central review of our cases and the literature showed that H3K27M-mutant EPN_PFA were similar to their counterparts with an EZHIP overexpression [8, 15]. We confirmed that EPN_PFA H3K27M-mutant are mainly classified within the subgroup 1f using DNA-methylation profiling, as previously reported [15].

In summary, H3K27M-mutant EPN_PFA may be a challenging diagnosis considering the pitfall of DMG with ependymal features, having important therapeutic and care support consequences. Tumor location, diffuse *versus* circumscribed tumoral architecture, Olig2 expression, type of histone gene mutations and DNA-methylation profiling must be considered in the integrated diagnosis.

## Supplementary Information

**Additional file 1: Figure 1**. Copy number profiles of cases of the cohort.

**Additional file 2: Table 1**. Summary of clinicopathological features of the H3K27M-mutant diffuse midline gliomas with ependymal tumor patients of the current study.
