# Supplementary material for: Posterior fossa ependymoma H3 K27-mutant: an integrated radiological and histomolecular tumor analysis
Source: Acta Neuropathol Commun. 2022 Sep 14;10:137. doi: 10.1186/s40478-022-01442-4 (PMC9476256; doi:10.1186/s40478-022-01442-4)

Case #1

Copy number prediction

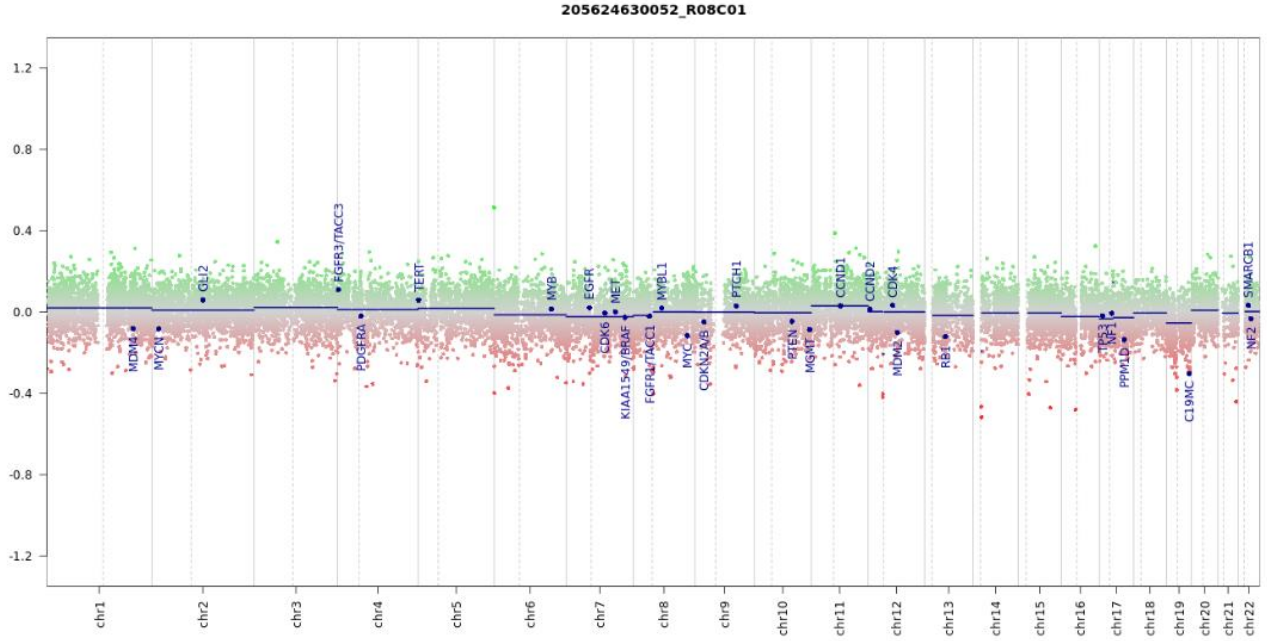

Case #2

Copy number prediction

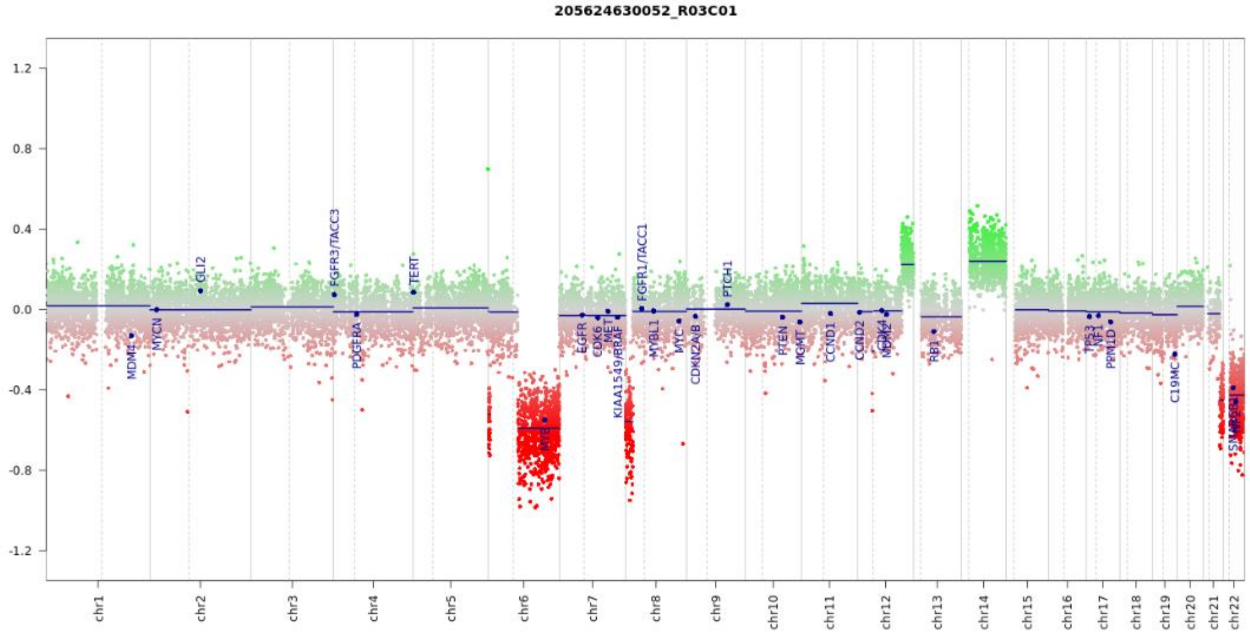

Case #3

Copy number prediction

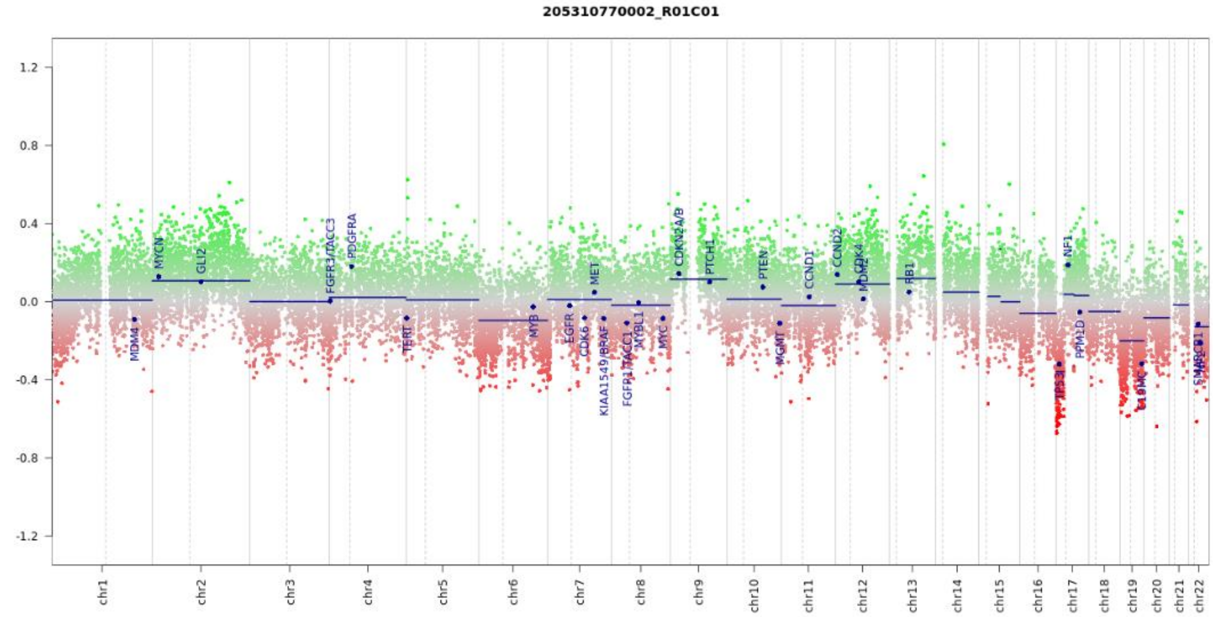

Case #4

Copy number prediction

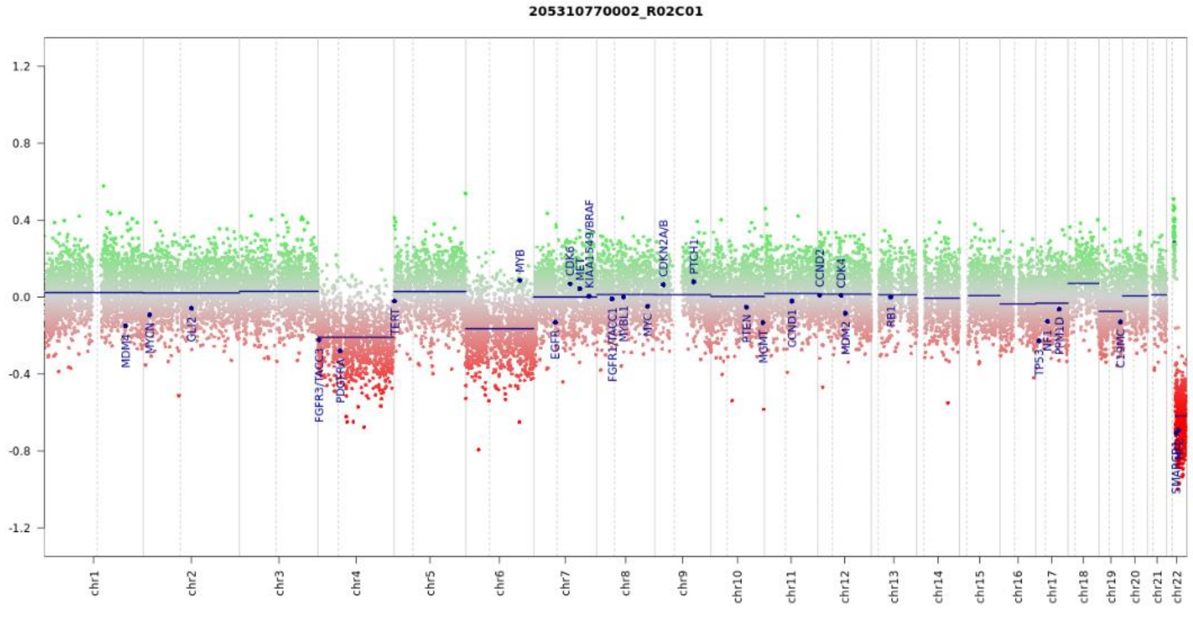

Case #5

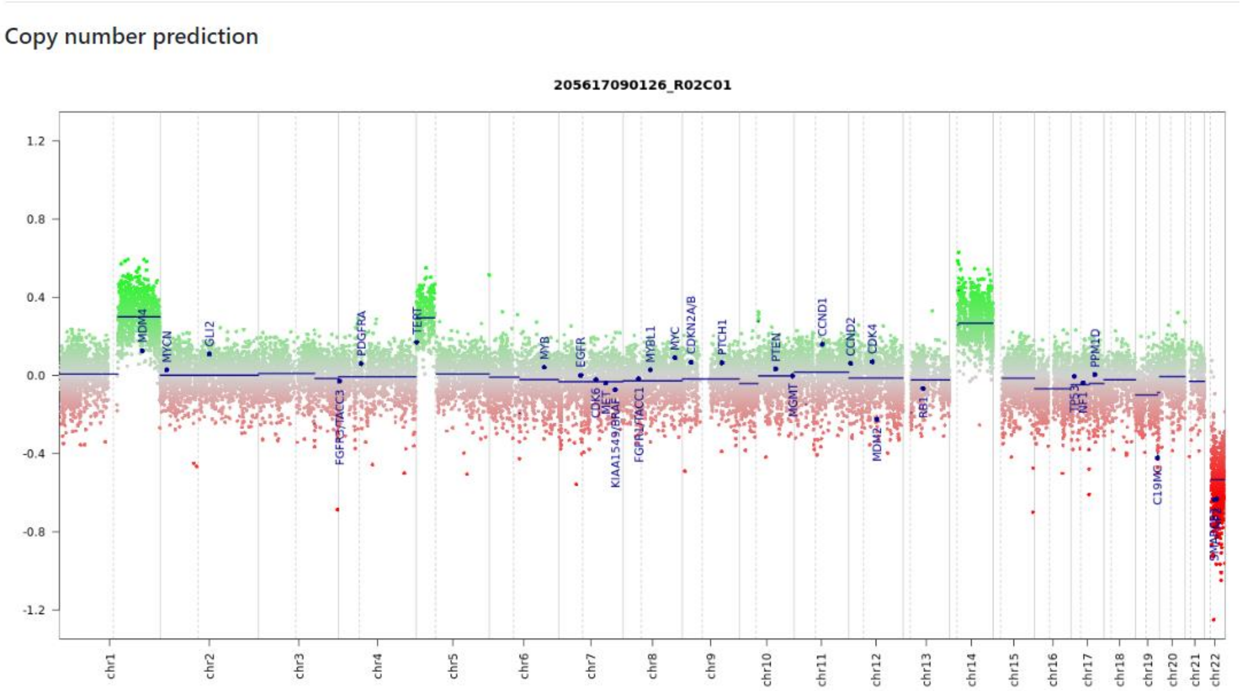
Copy number prediction
205617090126_R02C01
1.2
0.8
0.4
0.0
-0.4
-0.8
-1.2
chr1
chr2
chr3
chr4
chr5
chr6
chr7
chr8
chr9
chr10
chr11
chr12
chr13
chr14
chr15
chr16
chr17
chr18
chr19
chr20
chr21
chr22
MDM4
MYCN
GLI2
FGFR3/TACC3
PDGFRA
TERT
MYB
EGFR
CDK6
MET
KIAA1549/BRAF
FGFR1/TACC1
MYBL1
MYC
CDKN2A/B
PTCH1
PTEN
MGMT
CCND1
CCND2
CDK4
MDM2
RB1
TP53
NF1
PPM1D
C19MC

Case #6

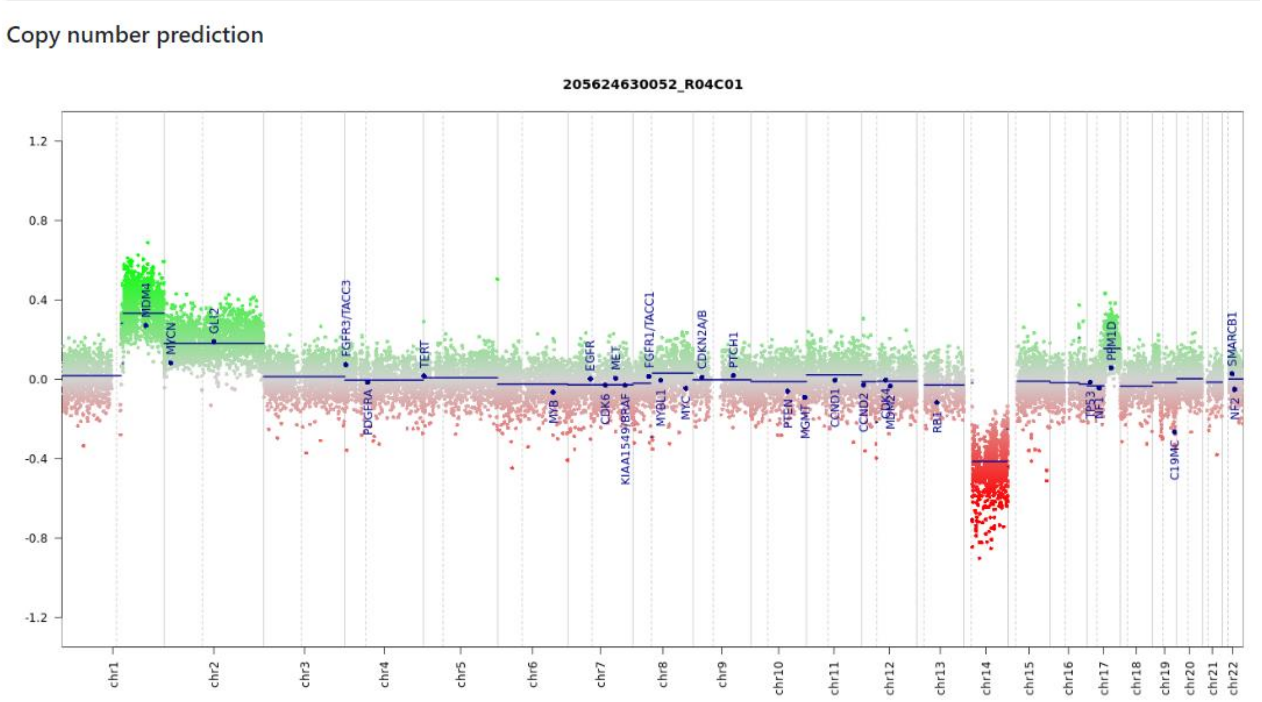
Copy number prediction
205624630052_R04C01
1.2
0.8
0.4
0.0
-0.4
-0.8
-1.2
chr1
chr2
chr3
chr4
chr5
chr6
chr7
chr8
chr9
chr10
chr11
chr12
chr13
chr14
chr15
chr16
chr17
chr18
chr19
chr20
chr21
chr22
MDM4
MYCN
GLI2
FGFR3/TACC3
PDGFRA
TERT
MYB
EGFR
CDK6
MET
KIAA1549/BRAF
FGFR1/TACC1
MYBL1
MYC
CDKN2A/B
PTCH1
PTEN
MGMT
CCND1
CCND2
CDK4
MDM2
RB1
TP53
NF1
PPM1D
C19MC
SMARCB1
NF2

Case #7

Copy number prediction

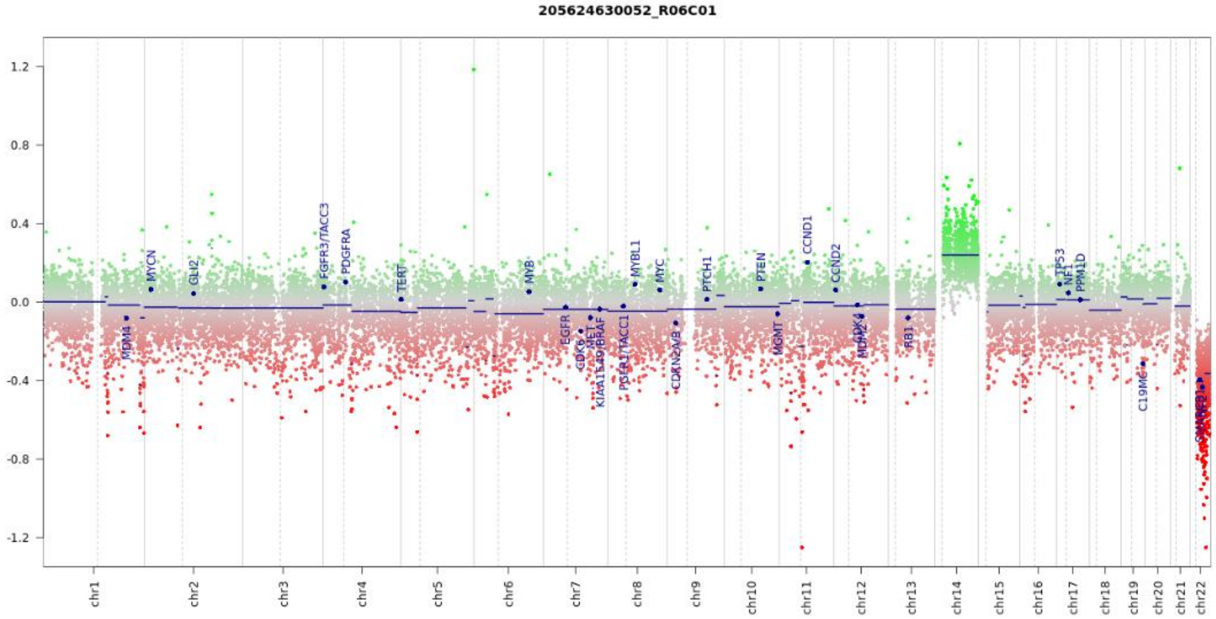

Case #8

Copy number prediction

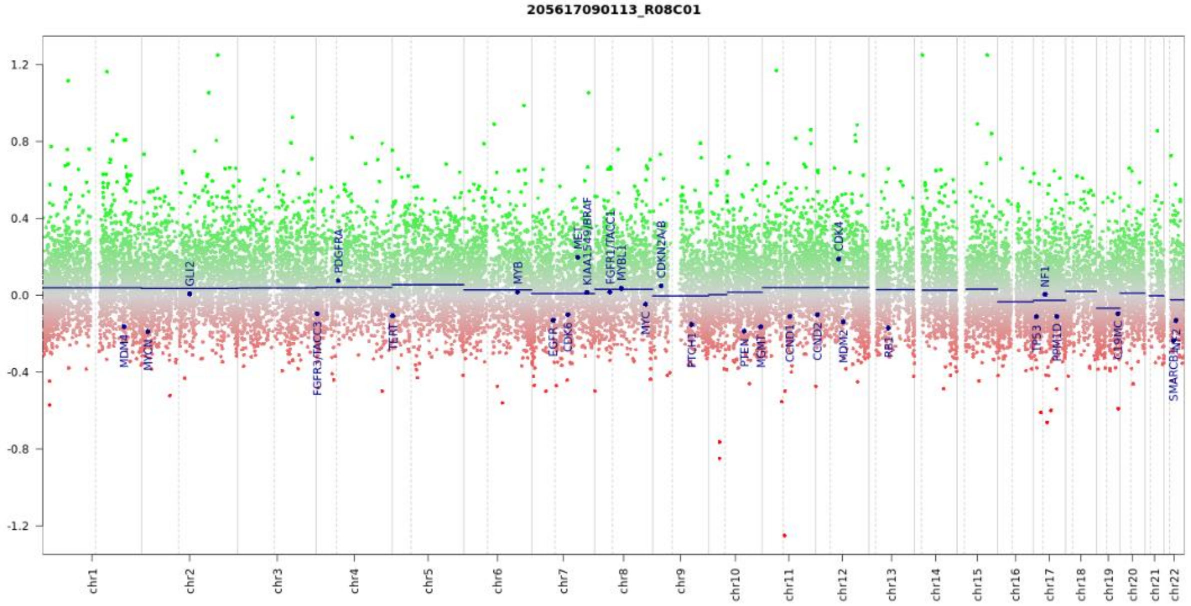

Case #9

Copy number prediction

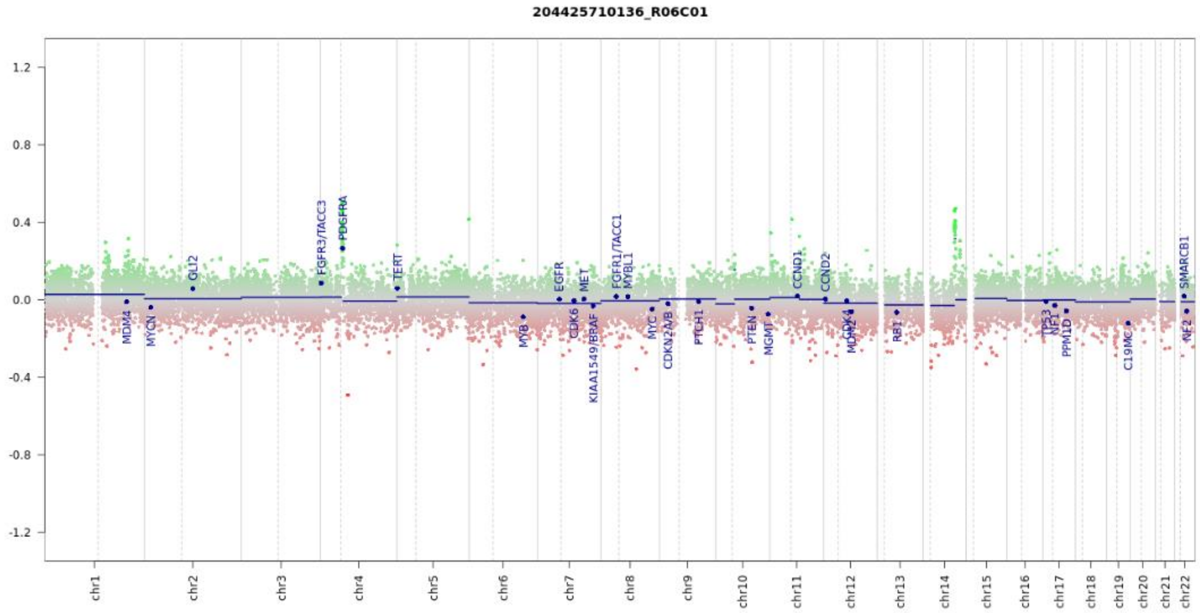

Supplement: Supplementary file 1 — Additional file 1: Figure 1. Copy number profiles of cases of the cohort. [file 40478_2022_1442_MOESM1_ESM.pdf]
