# Supplementary material for: Posterior fossa ependymoma H3 K27-mutant: an integrated radiological and histomolecular tumor analysis
Source: Acta Neuropathol Commun. 2022 Sep 14;10:137. doi: 10.1186/s40478-022-01442-4 (PMC9476256; doi:10.1186/s40478-022-01442-4)
Supplement: Supplementary file 2 — Additional file 2: Table 1. Summary of clinicopathological features of the H3K27M-mutant diffuse midline gliomas with ependymal tumor patients of the current study. [file 40478_2022_1442_MOESM2_ESM.docx]

|  | **Age (years) / Sex** | **WHO Grade** | **Mutation** | **1q gain/6q loss (CNV)** | **Methylation class (calibrated score)** | **Integrated diagnosis** | **Resection** | **Adjuvant therapy** | **Outcome** |
| --- | --- | --- | --- | --- | --- | --- | --- | --- | --- |
| 6 | 7 / M | ND | *H3F3A* | Present/absent | DMG H3K27M (0.84) | DMG, H3K27M | Biopsy | Radiotherapy Dasatinib  Bevacizumab | Death at 11 months |
| 9 | 8 / M | ND | *H3F3A* | Absent/absent | DMG H3K27M (0.54) | No | Biopsy | Radiotherapy Everolimus Bevacizumab | Death at 8 months |

Suppl. Table 1 Summary of clinicopathological features of the H3K27M-mutant diffuse midline gliomas with ependymal tumor patients of the current study

CNV: copy number variation; DMG: diffuse midline glioma; M: male; ND: not detailed; WHO: World Health Organization.
